# Identifying clinical phenotypes of frontotemporal dementia in post-9/11 era veterans using natural language processing

**DOI:** 10.3389/fneur.2024.1270688

**Published:** 2024-02-15

**Authors:** Samin Panahi, Jamie Mayo, Eamonn Kennedy, Lee Christensen, Sreekanth Kamineni, Hari Krishna Raju Sagiraju, Tyler Cooper, David F. Tate, Randall Rupper, Mary Jo Pugh

**Affiliations:** ^1^VA Salt Lake City Health Care System, Informatics, Decision-Enhancement and Analytic Sciences Center, Salt Lake City, UT, United States; ^2^Division of Epidemiology, University of Utah School of Medicine, Salt Lake City, UT, United States; ^3^Preventive Oncology, All India Institute of Medical Sciences, New Delhi, India; ^4^VA Salt Lake City Health Care System, Geriatric Research, Education and Clinical Center, Salt Lake City, UT, United States

**Keywords:** military health, frontotemporal dementia, phenotyping, veterans, natural language processing, traumatic brain injury

## Abstract

**Introduction:**

Frontotemporal dementia (FTD) encompasses a clinically and pathologically diverse group of neurodegenerative disorders, yet little work has quantified the unique phenotypic clinical presentations of FTD among post-9/11 era veterans. To identify phenotypes of FTD using natural language processing (NLP) aided medical chart reviews of post-9/11 era U.S. military Veterans diagnosed with FTD in Veterans Health Administration care.

**Methods:**

A medical record chart review of clinician/provider notes was conducted using a Natural Language Processing (NLP) tool, which extracted features related to cognitive dysfunction. NLP features were further organized into seven Research Domain Criteria Initiative (RDoC) domains, which were clustered to identify distinct phenotypes.

**Results:**

Veterans with FTD were more likely to have notes that reflected the RDoC domains, with cognitive and positive valence domains showing the greatest difference across groups. Clustering of domains identified three symptom phenotypes agnostic to time of an individual having FTD, categorized as Low (16.4%), Moderate (69.2%), and High (14.5%) distress. Comparison across distress groups showed significant differences in physical and psychological characteristics, particularly prior history of head injury, insomnia, cardiac issues, anxiety, and alcohol misuse. The clustering result within the FTD group demonstrated a phenotype variant that exhibited a combination of language and behavioral symptoms. This phenotype presented with manifestations indicative of both language-related impairments and behavioral changes, showcasing the coexistence of features from both domains within the same individual.

**Discussion:**

This study suggests FTD also presents across a continuum of severity and symptom distress, both within and across variants. The intensity of distress evident in clinical notes tends to cluster with more co-occurring conditions. This examination of phenotypic heterogeneity in clinical notes indicates that sensitivity to FTD diagnosis may be correlated to overall symptom distress, and future work incorporating NLP and phenotyping may help promote strategies for early detection of FTD.

## Introduction

Frontotemporal dementia (FTD) is a type of dementia that primarily affects the frontal and temporal lobes of the brain, leading to the progressive deterioration of behavior, personality, language abilities and executive dysfunction (1, 2). After Alzheimer’s disease (AD), FTD is the second most common cause of early-onset dementia (3). Unlike Alzheimer’s, which generally affects older individuals, FTD typically strikes at a younger age, with most cases occurring between 45 and 64 years of age (4). TBI and PTSD are both associated with an increased risk for neurodegenerative disorders, including FTD (5). Post-9/11 veterans represent a unique population with significant exposure to risk factors for FTD, as they are relatively young population and have a high prevalence of Traumatic Brain Injury (TBI) and Post-Traumatic Stress Disorder (PTSD) due to their military service experiences. The complexity of FTD presentation creates challenges for early detection, diagnosis, and treatment in this population.

FTD can manifest in two distinct clinical presentations; the behavioral variant of FTD (bvFTD) and the language variant (lvFTD). The behavioral variant is often marked by noticeable early-onset behavioral and by executive symptoms (2). The language variant is further classified into the semantic and non-fluent presentations of primary progressive aphasia (6). The behavioral and language signs and symptoms of FTD, however, often overlap in complex ways, and each individual symptom exists along a spectrum of severity. This makes FTD diagnosis challenging, and it is often misdiagnosed as a psychiatric disorder or stroke during the early stages (7). Therefore, an examination of the phenotypic heterogeneity of the disease is needed to improve identification. Clarifying the boundaries of FTD’s various presentations could also help clinician’s discriminate FTD from psychiatric disorders and stroke.

Identifying distinct phenotypes, defined as ‘any traits or characteristics that distinguish a specific state ‘could help to elucidate the heterogeneity of FTD case presentations (8). As FTD is heterogenous, FTD phenotyping demands particularly rich forms of data capable of discriminating subtle variations in FTD presentation. Natural Language Processing (NLP) presents a promising approach to phenotyping FTD because NLP can extract rich information from clinical notes across a patient’s full medical history (9). Mature NLP tools can automate the extraction of valuable information about the symptomology and characteristics of FTD, which may not be evident in traditional structured data (10). In this study, NLP tools were used to identify and characterize FTD-related symptoms and features in patient’s clinical notes. These features were then used to compare the histories of FTD cases to matched controls, and cluster distinct presentations within FTD cases.

## Materials and methods

### Cohort development

**Figure 1:**
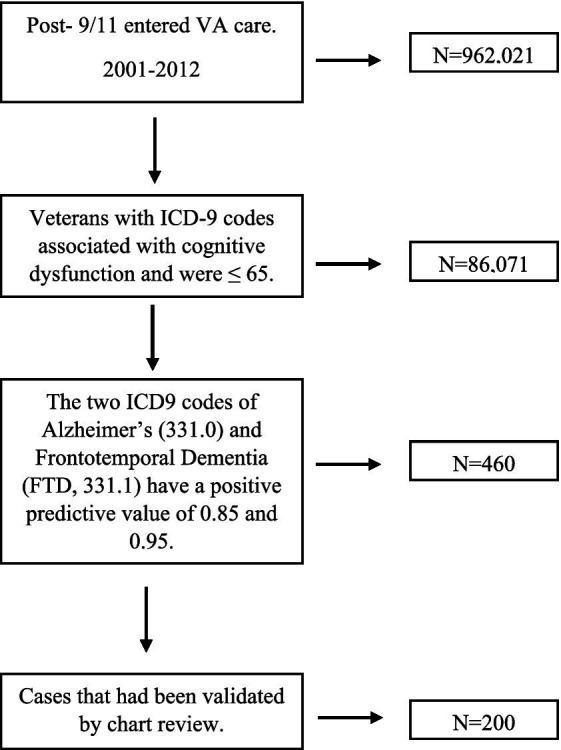
Cohort development flow chart diagram.

We initially identified post-9/11 era veterans who entered VA care between 2001–2012, had three or more years of VA care through the end of 2015, with one of those years being after 2007. Of those veterans, through 2019, *n* = 86,960 had an ICD9 code associated with cognitive dysfunction. Of the 86,960 patients identified, 98.98% of these cases (*n* = 86,071) were 65 years old or younger at the time of diagnosis. Those >65 years of age at the time of diagnosis were excluded. Because some of the ICD9 codes associated with cognitive dysfunction have poor predictive value in a population that is 65 years old or younger at the time of diagnosis (11). we only included those with ICD9 codes with a positive predictive value higher than 0.8 or that had been verified through expert chart review previously (11). The two ICD9 codes of Alzheimer’s (331.0) and Frontotemporal Dementia (FTD, 331.1) have a positive predictive value of 0.85 and 0.95, respectively, in a younger population (11). Our approach, then, was to consider those with an Alzheimer’s or FTD diagnosis positively validated (*n* = 460). We had 239 cases that had been validated by expert chart review in our previous study (11). This gave us a total of 699 cases that we treated as gold standards for training of our NLP system (Moonstone) (10). Since, the primary objective of our research was to discern whether it is possible to identify patients with TBI and cognitive dysfunction who are at heightened risk for developing FTD, it was essential to have a robust control group that mirrors the cases of interest in all respects. Therefore, cases were matched to at least one and up to four controls per case. Controls had to have a similar level of traumatic brain injury, if the case had a traumatic brain injury, but no indicator of cognitive dysfunction based on CTBIE and/or diagnosis codes. Cases were matched by age (± two years by birth year), gender, race, ethnicity, and year of first VA care. Nine of the cases of cognitive dysfunction lacked appropriate control matches, however, and were excluded leaving us with 690 cases and 2,624 control cases. We then randomly chose 200 FTD records with their matched controls (*n* = 713) for specific analysis of FTD (see Figure 1).

### Moonstone ontology/grammar rule building

The Moonstone NLP platform (10) is designed to extract data from clinical text not just by capturing explicitly stated information but also by inferring complex concepts often embedded in the nuanced language of common narrative. Moonstone diverges from typical NLP systems that require unambiguous phrasing, as it was originally developed to recognize social risk factors (SRF) like housing status, whether a patient lives alone, and the presence of social support. The ontology within Moonstone denotes a concept hierarchy that includes both literal and inferred instances—'patient in communication with family’ being a literal example, while ‘social support’ is more inferred (10).

Training of Moonstone for new NLP tasks involves the expansion of this ontology to encompass new concepts, supplemented by the creation of additional grammar rules until the system achieves satisfactory accuracy. For the purposes of our study, the ontology was augmented to include concepts pertaining to cognitive impairment, poor psychosocial function, and PTSD symptomatology. This enhancement was accomplished using two graphical tools: one for adding novel words and concepts to the ontology, and another for generating new grammar rules. This latter tool operates by allowing trainers to select from the array of “parse trees” that Moonstone produces when it processes sentences containing unknown words. From these trees, a new rule definition is extracted, to which a concept from the ontology is then attached. Consequently, this concept is applied to the interpretation of any phrase or sentence matching the new rule, thereby extending Moonstone’s analytical reach.

**Figure 2:**
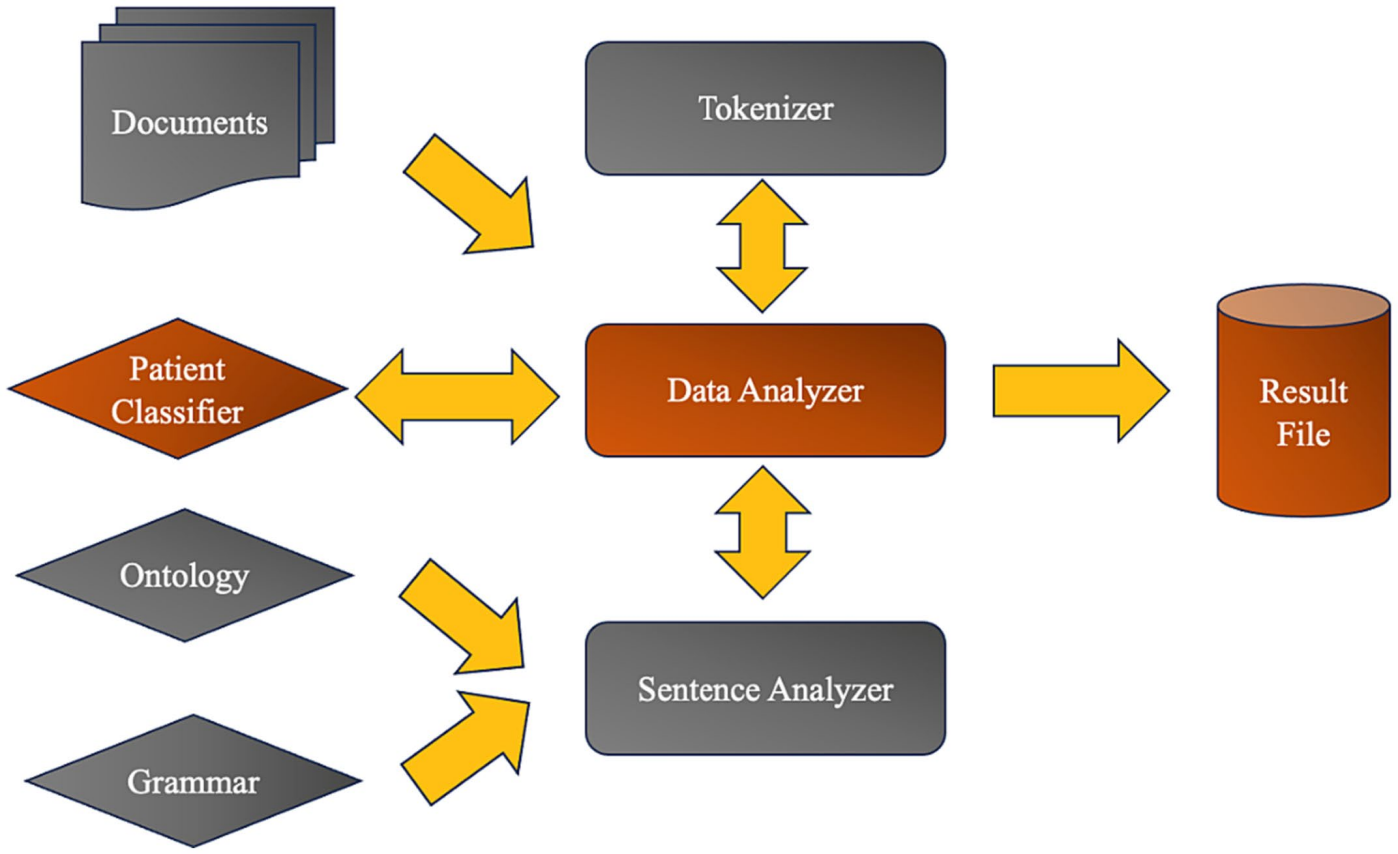
Moonstone system architecture.

The technique of expanding Moonstone’s capabilities was meticulously applied to sentences from a set of reports, which were utilized to train the platform for this project. Through this iterative process, Moonstone’s utility was refined, enabling it to more accurately parse and understand the complexities of clinical narratives related to cognitive and psychological assessments. After the ontology and grammar rule enhancement, and upon training Moonstone with the validated clinical notes, we employed a random forest classifier to identify cases with cognitive dysfunction. The classifier demonstrated a high level of precision, accurately identifying cases with an 88% success rate, further confirming the supervised nature of the learning paradigm employed by Moonstone (see Figure 2).

### Clinical note type selection and training

Ontology and grammar rule building in Moonstone was trained with manual text annotation of clinical notes from 165 cases of cognitive dysfunction validated by chart review and consensus of three neuropsychologists in a prior study and which were considered “gold standard” for this work. All the gold standard cases had neuropsychologist consult notes and neuroimaging notes. Annotators reviewed 15,985 note title types that existed in the electronic health record for the 165 gold standard cases and determined the most relevant note types for FTD. Annotators chose 3,108 note types to review for possible inclusion. Two nurse practitioners reviewed and validated the clinical text of 20% of these 3,108 note types and determined 1,195 note title types for inclusion in this study for training the NLP software, Moonstone (10). The annotators validated these notes for sentence level evidence of cognitive dysfunction, poor psychosocial function, and PTSD symptomology, and symptoms relevant to traumatic brain injury. Then based on the ontology lexicon, Moonstone read the clinical text and counted the number of times each concept was found in each patient’s history. Overall, 39 unique FTD-related concepts were identified by this process. Supplementary Table S1 provides a list of all 39 concepts.

### RDoC domain

The 39 NLP-derived ontologies were grouped into Research Domain Criteria (RDoC) domains to improve interpretation. The RDoC framework is a comprehensive approach developed by the National Institute of Mental Health (NIMH) to understand mental disorders based on underlying neurobiological and behavioral dimensions (12). The RDoC framework consists of multiple domains that capture the fundamental dimensions of human functioning and psychopathology. These domains are Cognitive, Positive Valence, Negative Valence, Social Processes, Sensorimotor, Arousal-Regulatory (12). In this work we additionally added the domain of “Interpersonal Trauma” to increase specificity to some underlying PTSD ontologies. To translate individual’s count of ontologies into a presence/absence of RDoC domains, we defined a domain as present in an individual’s records if at least half of the domain’s underlying ontologies were present in clinical notes.

### Comorbidities

Comorbidities were selected based on the most common medical conditions that can coexist alongside the FTD diagnosis including psychiatric disorders (e.g., Depression, anxiety, bipolar disorder), physical conditions (e.g., Stroke, hypertension, diabetics, Headache) (13–18).

## Analysis

### Statistical analysis

All analyses were scripted in Python 3. We used Z tests for univariate analyses to test differences in proportions between FTD and Controls using the statsmodels feature.

### Clustering

For clustering and dimensionality reduction, we employed the Uniform Manifold Approximation and Projection (UMAP) technique, utilizing frequency of 39 concepts extracted from the medical notes of our entire sample. UMAP is a manifold learning approach that facilitates the reduction of dimensions in the dataset. One of its key advantages lies in its ability to effectively preserve the global structure of high-dimensional data while simultaneously retaining the inter-sample distances. The subsequent clusters resulting from the UMAP-based analysis were assessed using the silhouette score method, a statistical measure that evaluates the effectiveness of a clustering technique by considering the defined subgroups’ quality in relation to their number (19).

### Word cloud

Word clouds are qualitative tools for visualizing the relative frequency of terms in text. Word cloud generating software was used to represent the relative frequency of symptom ontologies. The word cloud provides a summary overview of the most frequent FTD-specific concepts occurring in the medical notes for those with FTD relative to controls. In the word cloud, symptom ontologies that were more common in the FTD group are larger, and symptom ontologies that were equal across the two groups are represented in smaller text.

## Results

### Data summary

Table 1 presents sociodemographic and military measures for FTD cases and matched controls. After matching, the groups were statistically similar in terms of age, gender, education, race, ethnicity, and marital status, and military branch affiliations (*p* > 0.05). A significant difference emerged in the distribution of military rank, with the FTD group having a lower proportion of enlisted (*p* < 0.001) and a higher proportion of officers (*p* < 0.01).

**Table 1:** Sociodemographic and military measures for FTD cases and matched controls.

Group	FTD	Control	*p*
**Sample size (*n*)**	200	713	
**Age:** mean years	56.0	54.9	*>0.05*
Mean age at 1st dementia dx	47.8	–	–
> 65 years	25.5%	22.2%	*>0.05*
**Sex:** male	89.0%	90.2%	*>0.05*
Female	11.0%	9.8%	*>0.05*
**Education:** high school	47.3%	52.1%	*>0.05*
Some college +	38.2%	42.5%	*>0.05*
**Race:** Black	15.5%	16.4%	*>0.05*
White	71.5%	72.7%	*>0.05*
Other	13.0%	10.9%	*>0.05*
**Ethnicity:** Hispanic/Latino	11.0%	10.7%	*>0.05*
**Married/partnered:** yes	62.0%	62.8%	*>0.05*
**Military branch:** army	62.0%	67.6%	*>0.05*
Marine corps	11.0%	8.1%	*>0.05*
Air force	15.5%	13.8%	*>0.05*
Navy	10.0%	9.5%	*>0.05*
**Rank:** enlisted	62.5%	75.7%	**<0.001**
Officer	13.5%	7.15%	**<0.01**
Others (warrant, unknown)	24%	17.11%	*>0.05*

Bold values are significant.

Table 2 compares the incidence of comorbidities between the FTD group and matched controls. The FTD group showed significantly higher rates of overdose, depression, bipolar disorder, schizophrenia, suicidal ideation/attempt, stroke/CVD, cardiac issues, and seizures (*p* < 0.001).

**Table 2:** Comorbidity prevalence for the FTD group and matched controls.

Group	FTD	Control	*p*
**Sample size (*n*)**	200	713	
**Substance misuse:** alcohol abuse	28.0%	28.8%	*>0.05*
Substance abuse disorder	35.5%	34.1%	*>0.05*
Overdose, ever	11.5%	4.9%	**<0.001**
**Mental health:** depression	68.0%	54.1%%	**<0.001**
PTSD	53.0%	56.2%	*>0.05*
Anxiety	46.0%	38.7%	*>0.05*
Bipolar disorder	26.0%	14.9%	**<0.001**
Schizophrenia	4.0%	0.8%	**<0.01**
Suicidal ideation/attempt	15.5%	8.4%	**<0.01**
**Physical health**: any TBI	43.5%	47.7%	*>0.05*
Headache	48.5%	35.8%	*>0.05*
Brain tumor	1.0%	0.4%	*>0.05*
Stroke/CVD	24.0%	3.6%	**<0.001**
Cardiac	22.0%	10.9%	**<0.001**
Obesity	37.0%	35.1%	*>0.05*
Hypertension	47.5%	40.5%	*>0.05*
Seizure, any	20.0%	2.5%	**<0.001**
Insomnia	33.5%	25.4%	**<0.05**

Bold values are significant.

### Group comparison of NLP features

Table 3 compares the percentage of FTD cases and controls with evidence of each RDoC domain criteria in clinical notes. All RDoC domains showed significant percentage differences between the control group and the FTD group. The FTD group showed significantly higher percentages of individuals meeting the criteria for the cognitive, positive valence, negative valence, social processes, sensorimotor, arousal-regulatory, and interpersonal trauma domains. The cognitive-related ontologies showed the strongest association with FTD.

**Table 3:** Percentage incidence of ontologies that fall into the RDoC domains for the control and FTD groups, with *p*-values testing for groups differences per domain.

RDoC domain	Control	FTD	*p*
Cognitive	35.3%	82.0%	<0.0001*
Positive valence	35.6%	71.0%	<0.0001*
Negative valence	8.0%	24.5%	<0.0001*
Social processes	23.7%	46.4%	<0.0001*
Sensorimotor	1.8%	6.5%	0.0004*
Arousal-regulatory	11.8%	20.5%	0.0015*
Interpersonal trauma	8.6%	13.5%	0.036*

* indicates significance at <0.05.

**Figure 3:**
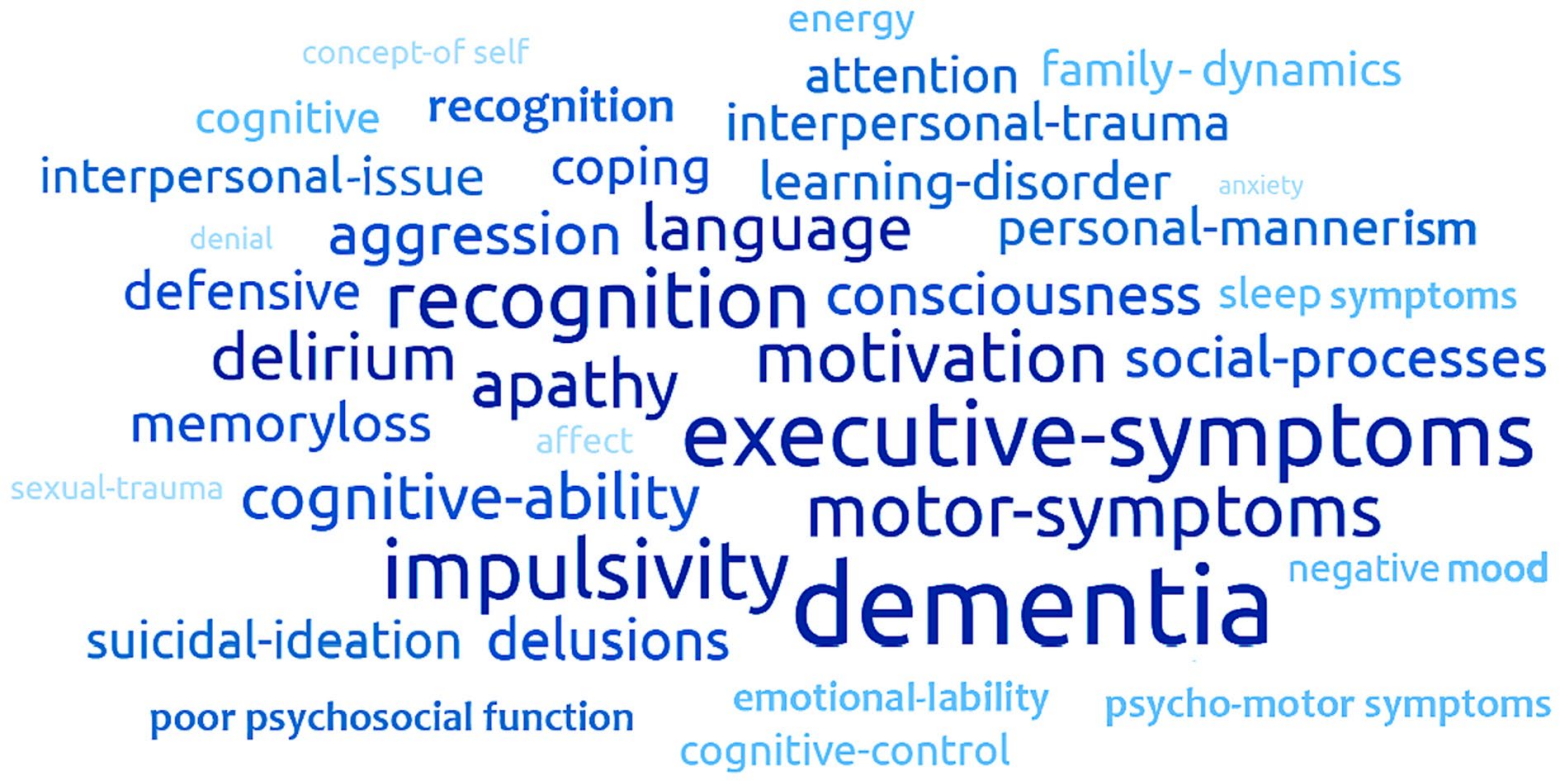
This figure provides a summary overview of the difference in words used in medical notes that were classified based on the FTD ontologies between patients with FTD and controls. For example, the largest words represent words that were classified by the ontologies far more frequently for those with FTD relative to controls. The smaller words represent concepts that were classified by the ontologies about the same frequency for people with FTD in relative to controls.

Figure 3 presents a word cloud of the most common behavioral characteristics among individuals with Frontotemporal Dementia (FTD) relative to matched controls. Dementia, impulsivity, executive symptoms, decision-making, and motor symptoms all featured prominently. A lack of recognition and motivation, alongside difficulties with social processes and interpersonal mannerisms featured moderately.

### Phenotypes of FTD

**Figure 4:**
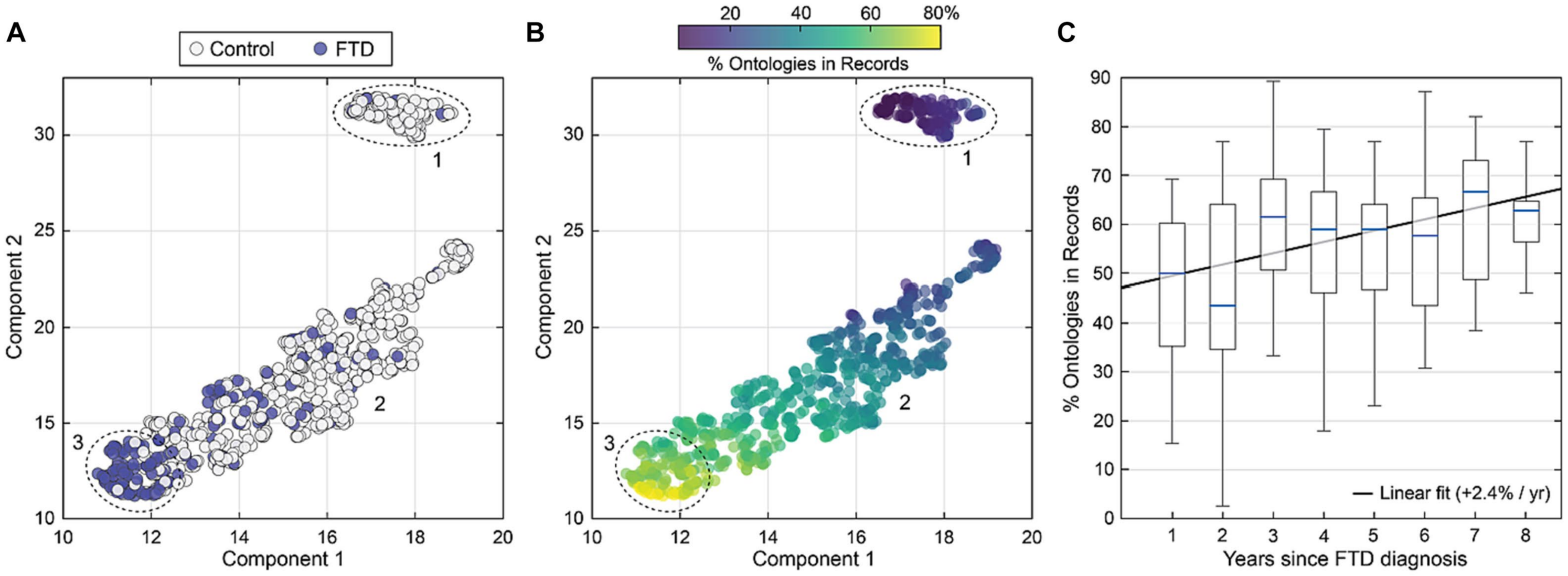
**(A)** A reduced dimensional representation of all sign/symptom ontologies is shown for all individuals, color coded by group membership (FTD, *n* = 200, blue circles. Controls, *n* = 713, white circles). Three regions showing individuals with similar symptomology are enumerated (1–3). **(B)** Like **(A)** showing the percentage of symptom ontologies present in clinical records per individual. Most FTD ontologies are present for those in region 3, whereas group 1 shows low rates of ontologies in records. **(C)** The percentage of all ontologies in records is shown as a function of time since first FTD diagnosis. Boxplots broken out per year indicate more FTD-related signs and symptoms in health records are evident for those with more time since first FTD diagnosis.

Figure 4 shows a two-dimensional representation of the seven RDoC domains produced using UMAP dimensional reduction (20). In Figure 4A, a UMAP dimensional reduction is shown color coded by group membership (FTD, n = 200, blue circles. Controls, n = 713, white circles) where the distance between points is a preserved estimate of the distance between individuals across all RDoC domains. In Figure 4B, the average percentage of all ontologies present in notes is shown against the percentage of veterans with FTD. Both measures were derived by iterating a boundary of inclusion across the ontology space in Figure 4C. There is a strong positive correlation between percentage with FTD and frequency of sign/symptom ontologies in clinical notes. For example, given a cluster where 70% are FTD+, then 71% of the 39 ontologies are present on average in clinical notes.

Table 4 represents the incidence of demographic and clinical characteristics across three phenotypes identified by the clustering approach (see method section): low distress (*N* = 149), moderate distress (*N* = 632), and high distress (*N* = 132). High distress individuals had a significantly higher incidence of FTD, 71.97% compared to 8.05 and 14.71% in the low and moderate distress groups, respectively (*p* < 0.001). Similar patterns were observed with total behavioral symptoms and various clinical characteristics like traumatic brain injury (TBI), cardiac issues, insomnia, obesity, stroke, headache, and seizures, with all showing a significantly higher prevalence in the high distress group (*p* < 0.001). Clinical conditions like schizophrenia, anxiety, bipolar disorder, depression, PTSD, overdose, substance abuse disorder, alcohol abuse, and suicide showed significantly higher incidence rates in the high distress group (*p* < 0.001). The average age was significantly lower in the high and moderate distress groups, and there were differences in racial distribution, with significantly more Hispanic and Black individuals in the high distress group.

**Table 4:** Percentage incidence of demographic and clinical characteristics criteria by each phenotype group.

	Low distress *N* = 149	Moderate distress *N* = 632	High distress *N* = 132	*p*-value
FTD	8.05%	14.71%	71.97%	<0.001
Total behavioral symptoms	8.9%	40%	67.3%	<0.001
Demographic characteristics				
Age	59.6 (9.8)	54.4 (10.6)	53.5 (10.4)	<0.001
Female	12.08%	9.97%	8.33%	>0.05
Race				
White	75.16%	71.83%	71.96%	>0.05
Hispanic	14.76%	8.38%	12.12%	0.006
Black	8.05%	18.35%	15.15%	0.008
Education				
College or more	58.33%	39.76%	34.37%	>0.05
Clinical characteristics				
Physical				
Any TBI	12.8%	50.6%	66.7%	<0.0001
Cardiac	10.7%	11.6%	25%	<0.0001
HBP	34.2%	43.4%	44.7%	0.10
Lung disease	9.4%	10.4%	12.1%	0.75
OSA	32.2%	35%	44.7%	0.06
Insomnia	15.4%	35%	44.7%	<0.0001
Obesity	31.5%	33.7%	48.5%	0.002
Stroke	3.4%	7.1%	18.2%	<0.001
Headache	18.8%	38.8%	59.8%	<0.001
Seizures	1.3%	5.1%	18.2%	<0.001
Psychological				
schizophrenia	0.0%	1.09%	5.3%	0.0004
Anxiety	15.4%	42.69%	56.8%	0.0001
Bipolar	3.4%	16.9%	34.8%	0.0001
Depression	24.8%	60.9%	75.8%	<0.001
PTSD	18.8%	60.8%	72%	<0.001
Overdose	1.3%	5.1%	18.2%	<0.001
Substance abuse disorder	10.7%	36.9%	49.2%	<0.001
Alcohol abuse	9.4%	31%	38.6%	<0.0001
Suicide	0.0%	9.0%	25.8%	<0.0001

**Figure 5:**
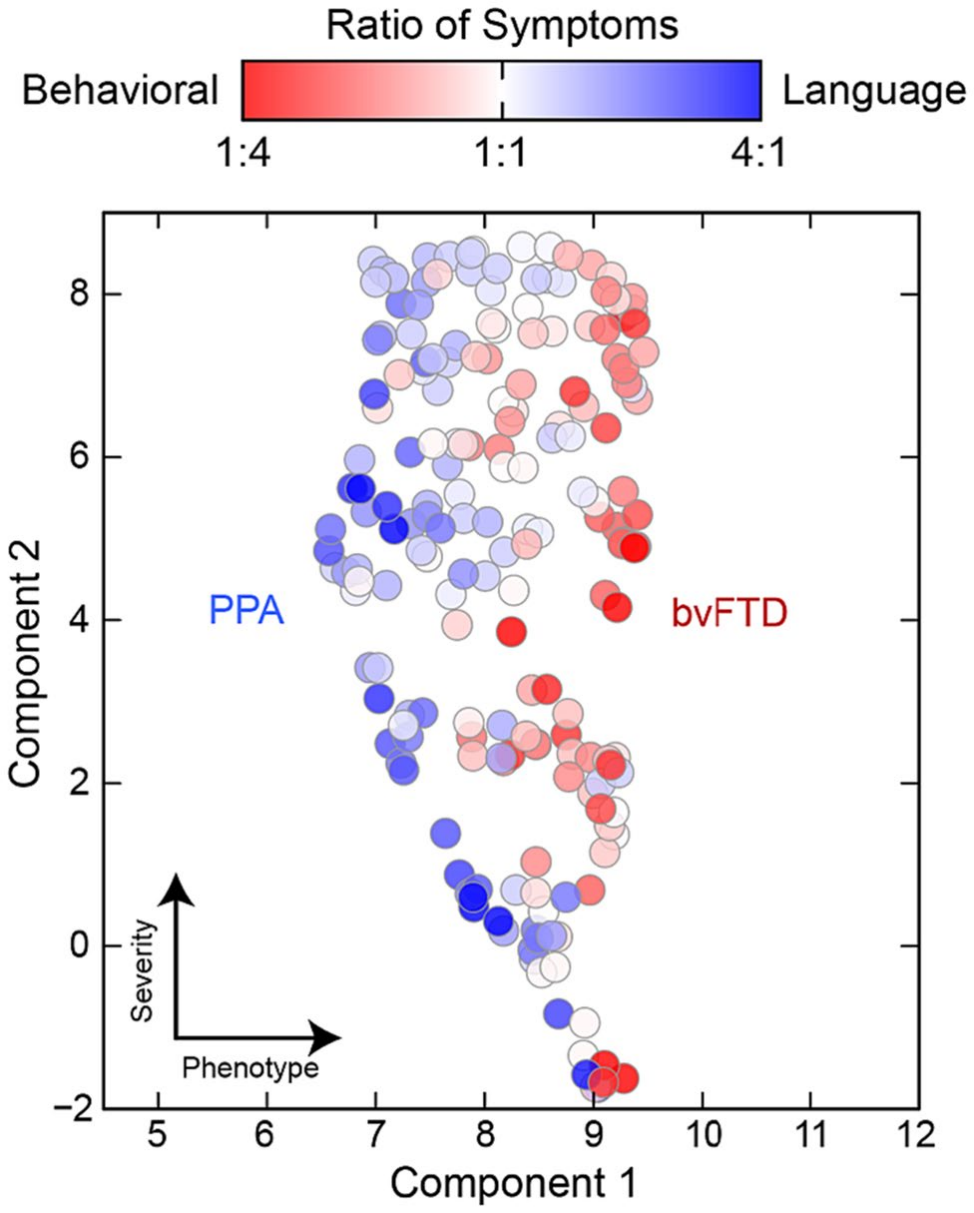
UMAP dimensional reduction of RDoC domains for the FTD+ group only (*n* = 200). To identify FTD variants, individuals were color coded by the relative ratio of behavioral (red) to language (blue) related concepts in their clinical notes. Colored clusters indicate individuals presenting with distinct behavioral and language variants and symptomology.

Figure 5 assess whether distinct subtypes are identified through clustering. The UMAP dimensional reduction of RDoC domains was performed specifically for the FTD group, comprising 200 cases, resulting in a 2D ‘symptom space’. Next, two indices were created: (1) Behavioral concepts (e.g., impulsivity, disinhibition, apathy, and behavioral traits), and (2) Language concepts (e.g., language, speech, learning, executive functions, and memory). The ratio of these two symptom sets was calculated for each individual, and a color code was assigned based on the ratio: records with more behavioral symptoms were marked as RED, while those with more language-related issues in text notes were labeled BLUE. Subsequently, the distribution of these color-coded ratios was evaluated across the RDoC space, where clustering of colors would indicate the presence of subtypes.

## Discussion

In this study, NLP-aided medical chart reviews successfully identified distinct phenotypes of FTD and provided a novel signature of RDoC domain distress. Prior research has leveraged unsupervised learning and clustering approaches applied to dementia cohorts. These include clusters of cognitive impairment using biomarkers, anatomical cluster identification and genetic variant mapping, although no clustering studies have specifically evaluated post-9/11 era veterans with FTD (8, 21). Our findings align with prior work by demonstrating the presence of distinct phenotypes within the FTD group, as evidenced by the clustering of clinical text features. The identification of Low, Moderate, and High distress phenotypes in our study expands upon prior work and provides further evidence for the existence of diverse clinical subgroups within FTD.

The diagnosis of Early onset FTD poses challenges due to its relative rarity, and its highly variable clinical manifestations that can mirror psychiatric disorders and neurological conditions such as stroke (2). The FTD diagnostic process is further complicated by the phenotypic heterogeneity of FTD, which encompasses many distinct behaviors, affective changes, and movement and speech difficulties. NLP provides an appropriate framework to capture these complex patterns, because NLP tools can glean valuable information about subtle features buried within a large corpus of clinical text, far beyond the simple presence/absence encodings typically found in health systems data. Future work may benefit from the use of NLP phenotyping pipelines trained on FTD-specific text features.

To facilitate clinical intuition, raw NLP ontologies extracted from text were organized into validated RDoC domains. RDoC domains were then clustered into a low dimensional space to enable visualization and the identification of three distinct phenotypes (Low, Moderate, and High distress). This analysis revealed a continuum of distress within and across FTD variants, with some diagnosed FTD cases showing surprisingly low levels of symptom distress, although the majority were in the Moderate to High groups. This approach demonstrates how unstructured clinical text can be used to assess the heterogeneity of neurological disease. Future work could include an in-depth temporal analysis to better understand how time from diagnosis influences our current model of symptom distress and how different phenotypes progress through the disease over time.

A comparison of the FTD group and matched controls revealed large differences in the incidence of multiple comorbidities. Prior work has found links between military related TBI and PTSD and FTD (22). The strong associations with specific comorbidities and FTD found in this study reinforce these connections. These findings have implications for identification and care, as these individuals present with a degree medical complexity that demands detailed and appropriate treatment strategies. Additionally, The FTD group exhibited significantly higher rates of overdose, depression, bipolar disorder, schizophrenia, suicidal ideation/attempt, stroke/CVD, cardiac issues, and seizures. FTD is associated with a higher burden of psychiatric and neurological comorbidities which may contribute to the complexity of its clinical presentation as demonstrated by the high prevalence of comorbidities identified among those with FTD. Thus, the broader clinical context is crucial when evaluating individuals for FTD, as the presence of these comorbidities may influence disease progression and treatment efficacy. A limitation of the interpretation of this data is a lack of review of the validity of psychiatric diagnosis associated with the FTD cases. For example, a patient could be misdiagnosed with bipolar disorder early on in the disease process, but then be diagnosed with FTD after consultation with experts and progression over time. It could be helpful for clinicians to continue to consider FTD as a rule out early on in the diagnostic stages, given the large overlap of FTD with psychiatric presentations.

Overall, those with FTD had higher risk of suicidal ideation and overdose as compared to controls and this could be an important factor when trying to decide on early intervention approaches and psychoeducation for clinicians and/or caregivers in the future studies. Additionally, the FTD cases in this study had the features of emotional liability and interpersonal trauma one might see in psychiatric disorders but this was often coupled with an impulsivity that could be associated with the high rates of overdose and interpersonal conflict. This is consistent with the current studies regarding FTD in the general population (23, 24). Future studies looking at the effectiveness of therapeutic and pharmacological approaches aimed at mitigating this impulsivity could help to inform treatment options across phenotypes in the future (24). Our NLP approach is limited in being able to differentiate between apathy and impulsivity, or even to consistently identify apathy, because it is reliant on clinical bias in reporting while note taking, but it can identify these concepts generally across a large population which could help to aid future studies.

From chart review and verified with NLP analysis across cases, FTD cases had significantly higher incidence of interpersonal trauma as compared to control, although controls in this population also had incidences of interpersonal trauma. For the cases that were chart reviewed, this interpersonal trauma was related to high reports of distress, substance use, and suicidal ideation. This is consistent with work done by Takeda et al. and Massimo et al. showing the impact of FTD on caregivers and the impact of FTD on relationships (25, 26). Our work is novel in that we were able to identify these issues from a large-scale NLP approach and validate these findings within our specific population. Future work could include studies evaluating the effectiveness of targeting therapeutic approaches aimed at helping people with FTD and their caregivers manage these interpersonal relationships and the difficulty of dealing with the relationship issues that arise given the stress of the disease could help in treatment of this disease.

In our statistical evaluation of symptoms over time since diagnosis, symptoms seemed to increase over time (Figure 3C). It is unclear, however, if this is due to lack of effective treatment or progression of the disease. Either way, taking the current literature as a whole, managing impulsivity and supporting patients in improving interpersonal relationships across the disease progression and across the lifespan, could be key in making a clinical impact on the experience of distress in this patient population.

Cumulative symptom severity across all domains distinguished FTD subtypes in important ways that may compliment the typical classification of FTD by variant. Our study explored the existence of distinct subtypes within the FTD population based on symptom presentation. By performing dimensional reduction of RDoC domains for the FTD group and creating two indices for Behavioral and Language variant, we assessed the variability in symptom profiles among veterans with FTD and were able to identify a unique subtype with distinct symptom profiles. Our result shows that phenotyping approaches may help to further elucidate the relationship between FTD symptom distress and disease progression, enabling more accurate prognoses. Future work could also explore whether an NLP tool for assessing overall dementia symptom severity could serve as a rapid heuristic for population level disease progression. Automated NLP screening of distress could also be useful for validating or extending existing tools such as the Frontotemporal dementia Rating Scale, FRS in large populations (27). This study highlights how clinical phenotyping and clustering approaches may offer opportunities to better understand rare and heterogeneous diseases and improve early detection and clinical care for individuals living with dementia.

### Limitation

This study, focused on identifying the clinical phenotypes of Frontotemporal Dementia (FTD) among post-9/11 era veterans, holds several limitations. The generalizability of results is restricted given the specific study demographic, while the retrospective design could introduce bias due to the potential for incomplete historical medical records. The study relies on ICD-10 codes for identifying FTD cases. The number of FTD cases is relatively small (*n* = 200), which might limit the statistical power of the study.

## Conclusion

This study demonstrated the potential of NLP and phenotyping approaches to enhance the classification of FTD subtypes, considering cumulative symptom severity alongside the traditional variant-based classification. By leveraging NLP and validated domains, valuable insights into distress levels, comorbidities, and interpersonal relationships in FTD patients were gained. The findings revealed that FTD exhibits a continuum of severity and symptom distress, both within and across variants, with distress levels often co-occurring with other conditions. This highlights the importance of sensitivity to overall symptom distress in diagnosing FTD and suggests that incorporating NLP and phenotyping methods could aid in early detection strategies for FTD, ultimately contributing to improved patient outcomes.

## Data availability statement

The data analyzed in this study is subject to the following licenses/restrictions: these data sets are part of the VA medical record system. Requests to access these datasets should be directed to maryjo.pugh@hsc.utah.edu.

## Ethics statement

The studies involving humans were approved by University of Utah and the Veterans Administration in Salt Lake City, Utah. The studies were conducted in accordance with the local legislation and institutional requirements. Written informed consent for participation was not required from the participants or the participants’ legal guardians/next of kin in accordance with the national legislation and institutional requirements.

## Author contributions

SP: Conceptualization, Methodology, Writing – review & editing, Data curation, Formal analysis, Visualization. JM: Conceptualization, Methodology, Writing – review & editing, Investigation, Project administration, Supervision, Writing – original draft. EK: Formal analysis, Visualization, Writing – review & editing. LC: Data curation, Software, Writing – review & editing. SK: Data curation, Methodology, Writing – review & editing. HS: Conceptualization, Funding acquisition, Methodology, Writing – review & editing. TC: Data curation, Writing – review & editing. DT: Writing – review & editing. RR: Writing – review & editing. MP: Writing – review & editing.

## References

[1] Lashley T Rohrer JD Mead S Revesz T. Review: an update on clinical, genetic and pathological aspects of frontotemporal lobar degenerations. Neuropathol Appl Neurobiol. (2015) 41:858–81. doi: 10.1111/nan.12250, PMID: 26041104

[2] Rascovsky K Hodges JR Knopman D Mendez MF Kramer JH Neuhaus J. Sensitivity of revised diagnostic criteria for the behavioural variant of frontotemporal dementia. Brain. (2011) 134:2456–77. doi: 10.1093/brain/awr179, PMID: 21810890 PMC3170532

[3] Ratnavalli E Brayne C Dawson K Hodges JR. The prevalence of frontotemporal dementia. Neurology. (2002) 58:1615–21. doi: 10.1212/wnl.58.11.1615 12058088

[4] Onyike CU Diehl-Schmid J. The epidemiology of frontotemporal dementia. Int Rev Psychiatry. (2013) 25:130–7. doi: 10.3109/09540261.2013.776523, PMID: 23611343 PMC3932112

[5] Gardner RC Yaffe K. Epidemiology of mild traumatic brain injury and neurodegenerative disease. Mol Cell Neurosci. (2015) 66(Pt B):75–80. doi: 10.1016/j.mcn.2015.03.001, PMID: 25748121 PMC4461453

[6] Gorno-Tempini ML Hillis AE Weintraub S Kertesz A Mendez M Cappa SF. Classification of primary progressive aphasia and its variants. Neurology. (2011) 76:1006–14. doi: 10.1212/WNL.0b013e31821103e6, PMID: 21325651 PMC3059138

[7] Galimberti D Dell'Osso B Altamura AC Scarpini E. Psychiatric symptoms in frontotemporal dementia: epidemiology, phenotypes, and differential diagnosis. Biol Psychiatry. (2015) 78:684–92. doi: 10.1016/j.biopsych.2015.03.028, PMID: 25958088

[8] Pugh MJ Kennedy E Prager EM Humpherys J Dams-O'Connor K Hack D. Phenotyping the Spectrum of traumatic brain injury: a review and pathway to standardization. J Neurotrauma. (2021) 38:3222–34. doi: 10.1089/neu.2021.0059, PMID: 33858210 PMC8917880

[9] Sheikhalishahi S Miotto R Dudley JT Lavelli A Rinaldi F Osmani V. Natural language processing of clinical notes on chronic diseases: systematic review. JMIR Med Inform. (2019) 7:e12239. doi: 10.2196/12239, PMID: 31066697 PMC6528438

[10] Conway M Keyhani S Christensen L South BR Vali M Walter LC. Moonstone: a novel natural language processing system for inferring social risk from clinical narratives. J Biomed Semantics. (2019) 10:6. doi: 10.1186/s13326-019-0198-0, PMID: 30975223 PMC6458709

[11] Marceaux JC Soble JR O'Rourke JJF Swan AA Wells M Amuan M. Validity of early-onset dementia diagnoses in VA electronic medical record administrative data. Clin Neuropsychol. (2020) 34:1175–89. doi: 10.1080/13854046.2019.1679889 31645200 PMC13157277

[12] Hakak-Zargar B Tamrakar A Voth T Sheikhi A Multani J Schütz CG. The utility of research domain criteria in diagnosis and Management of Dual Disorders: a Mini-review. Front Psych. (2022) 13:805163. doi: 10.3389/fpsyt.2022.805163, PMID: 35299823 PMC8923302

[13] Torralva T Sposato LA Riccio PM Gleichgerrcht E Roca M Toledo JB. Role of brain infarcts in behavioral variant frontotemporal dementia: Clinicopathological characterization in the National Alzheimer's coordinating center database. Neurobiol Aging. (2015) 36:2861–8. doi: 10.1016/j.neurobiolaging.2015.06.026, PMID: 26220367 PMC4562890

[14] Kuźma E Lourida I Moore SF Levine DA Ukoumunne OC Llewellyn DJ. Stroke and dementia risk: a systematic review and meta-analysis. Alzheimers Dement. (2018) 14:1416–26. doi: 10.1016/j.jalz.2018.06.3061, PMID: 30177276 PMC6231970

[15] Hagen K Stordal E Linde M Steiner TJ Zwart JA Stovner LJ. Headache as a risk factor for dementia: a prospective population-based study. Cephalalgia. (2014) 34:327–35. doi: 10.1177/0333102413513181 24265286

[16] Urban-Kowalczyk M Kasjaniuk M Śmigielski J Kotlicka-Antczak M. Major depression and onset of frontotemporal dementia. Neuropsychiatr Dis Treat. (2022) 18:2807–12. doi: 10.2147/NDT.S390385, PMID: 36471745 PMC9719411

[17] Gliebus G. A case report of anxiety disorder preceding frontotemporal dementia with asymmetric right temporal lobe atrophy. SAGE Open Med Case Rep. (2014) 2:2050313X13519977. doi: 10.1177/2050313X13519977, PMID: 27489637 PMC4857347

[18] Salzbrenner LS Brown J Hart G. Frontotemporal dementia complicated by comorbid borderline personality disorder: a case report. Psychiatry (Edgmont). (2009) 6:28–31., PMID: 19724729 PMC2714814

[19] McInnes L. Healy J. Melville J. (2018) UMAP: Uniform Manifold Approximation and Projection for Dimension Reduction. Available at: http://arxiv.org/abs/1802.03426

[20] Rousseeuw PJ Leroy AM. Robust regression and outlier detection Wiley Interscience, New York: Oxford university press (Series in Applied Probability and Statistics), (1987). 329 p.

[21] Gamberger D Lavrač N Srivatsa S Tanzi RE Doraiswamy PM. Identification of clusters of rapid and slow decliners among subjects at risk for Alzheimer’s disease. Sci Rep. (2017) 7:6763. doi: 10.1038/s41598-017-06624-y, PMID: 28755001 PMC5533731

[22] Whitwell JL Przybelski SA Weigand SD Ivnik RJ Vemuri P Gunter JL. Distinct anatomical subtypes of the behavioural variant of frontotemporal dementia: a cluster analysis study. Brain. (2009) 132:2932–46. doi: 10.1093/brain/awp232, PMID: 19762452 PMC2768663

[23] Kennedy E Panahi S Stewart IJ Tate DF Wilde EA Kenney K. Traumatic brain injury and early onset dementia in post 9-11 veterans. Brain Inj. (2022) 36:620–7. doi: 10.1080/02699052.2022.2033846, PMID: 35125061 PMC9187585

[24] Lansdall CJ Coyle-Gilchrist ITS Jones PS Vázquez Rodríguez P Wilcox A Wehmann E. Apathy and impulsivity in frontotemporal lobar degeneration syndromes. Brain. (2017) 140:1792–807. doi: 10.1093/brain/awx101, PMID: 28486594 PMC5868210

[25] Zucca M Rubino E Vacca A Govone F Gai A De Martino P. High risk of suicide in behavioral variant frontotemporal dementia. Am J Alzheimers Dis Other Dement. (2019) 34:265–71. doi: 10.1177/1533317518817609 PMC10852495 30558441

[26] Massimo L Evans LK Benner P. Caring for loved ones with frontotemporal degeneration: the lived experiences of spouses. Geriatr Nurs. (2013) 34:302–6. doi: 10.1016/j.gerinurse.2013.05.001, PMID: 23726759 PMC3867267

[27] Takeda A Sturm VE Rankin KP Ketelle R Miller BL Perry DC. Relationship turmoil and emotional empathy in frontotemporal dementia. Alzheimer Dis Assoc Disord. (2019) 33:260–5. doi: 10.1097/WAD.0000000000000317, PMID: 31135456 PMC6710105

