# Supplementary material for: Identifying clinical phenotypes of frontotemporal dementia in post-9/11 era veterans using natural language processing
Source: Front Neurol. 2024 Feb 15;15:1270688. doi: 10.3389/fneur.2024.1270688 (PMC10902457; doi:10.3389/fneur.2024.1270688)
Supplement: Supplementary file 1 [file Data_Sheet_1.pdf]

Supplement 1A: Concepts in the Ontology

1. Concept of self
2. cognitive
3. Interpersonal issue
4. denial
5. aggression
6. defensive
7. delirium
8. Memory loss
9. Sexual trauma
10. Cognitive ability
11. Suicidal ideation
12. Delusion
13. Poor psychosocial function
14. Impulsivity
15. affect
16. Apathy
17. Recognition
18. Aggression
19. Language
20. Coping
21. Recognition
22. Energy
23. Family dynamics
24. Interpersonal trauma
25. Personal mannerism
26. Sleep symptoms
27. Social processes
28. Executive symptoms
29. Motor symptoms
30. Negative mood
31. Dementia
32. Emotional liability
33. Cognitive control
34. Psycho motor symptoms
35. Attention
36. Anxiety
37. consciousness
38. motivation
39. learning- disorder

Supplement 1B: Glossary of Technical Terms

**NLP (Natural Language Processing):**	The technology enabling computers to understand and process human language, bridging the gap between human communication and computer understanding.
**Ontology**	A framework in NLP that organizes and defines the relationships between different concepts, aiding in the system's comprehension of context.
**Grammar Rule (in NLP systems):**	Instructions in an NLP system that define how to interpret the structure and meaning of sentences within a language.
